# Optimizing SPS-Processed Pure Tantalum: Effects of Temperature, Pressure, and Time

**DOI:** 10.3390/ma19030621

**Published:** 2026-02-05

**Authors:** Hui Huang, Chen Gong, Shihai Miao, Jiaqi Zhang, Yu Zhang, Xia Liu, Ying Li, Yibo Wei, Yafei Pan

**Affiliations:** 1 Shandong Non-Metal Materials Institute, Jinan 250031, China; 18854138881@163.com (C.G.); miaoshihai@126.com (S.M.);; 2 School of Materials Science and Engineering, Hefei University of Technology, Hefei 230009, China

**Keywords:** pure tantalum, spark plasma sintering, densification, microstructure, mechanical properties

## Abstract

Pure tantalum (Ta) is widely used in applications such as capacitors and semiconductor coatings due to its high melting point, excellent corrosion resistance, and good biocompatibility. In this study, spark plasma sintering (SPS) technology has been employed to successfully prepare high-density, fine-grained pure Ta through systematic optimization of sintering temperature, pressure, and holding time. The results indicate that sintering temperature plays a predominant role on the densification behavior. Increasing the sintering pressure and prolonging the holding time also contribute to further enhancing the densification. Under the process conditions of 1450 °C, 40 MPa, and a holding time of 10 min, the relative density of the sample reaches 98.7%. Microstructural analysis reveals that the sintering process of pure Ta can be divided into two main stages: densification-dominated and grain growth-dominated. When the relative density exceeds a threshold value (approximately 96% in this study), the grain size increases rapidly from 4.43 μm to 28.87 μm. This grain coarsening leads to a transition in the fracture mechanism from a mixed mode of intergranular and cleavage fractures to completely intergranular fracture, which significantly reduces the bending strength and plastic deformation capacity of the material.

## 1. Introduction

Tantalum (Ta), a refractory metal renowned for its exceptional properties including an extremely high melting point (3017 °C), outstanding corrosion resistance, good biocompatibility, and excellent dielectric strength, has become an indispensable strategic material in modern high-technology industries [1,2]. These superior characteristics enable Ta and its alloys to perform reliably in extreme environments involving high temperatures, high pressures, and aggressive chemicals [3,4].

The applications of Ta are primarily driven by its two key attributes. Firstly, its innate ability to form a stable, dense oxide film (Ta_2_O_5_) on its surface makes it the premier choice for solid electrolytic capacitors [5,6], which are critical components in virtually all electronic devices, from smartphones to aerospace systems, due to their high reliability and volumetric efficiency [7,8]. Secondly, its high melting point and sputtering performance under bombardment render pure Ta a high-quality sputtering target material for the physical vapor deposition (PVD) process [9,10], which is essential for creating thin-film coatings in semiconductor integrated circuits and advanced display panels [11,12].

The widespread utilization of Ta and related alloys heavily relies on effective and economical processing techniques. Conventional preparation methods mainly include electron beam melting (EBM) and powder metallurgy (PM). Electron beam melting can produce high-purity Ta and related-alloys but often results in coarse grains and significant chemical heterogeneity (e.g., microsegregation and macrosegregation) [13,14], which arises from solute redistribution induced by high thermal gradients and rapid solidification rates during the process [15,16]. Subsequent thermomechanical processing (forging, rolling) is therefore required not only to refine the coarse microstructure [17,18] but also to homogenize the chemically segregated structure, which inevitably increases both energy consumption and production costs [19,20].

While traditional powder metallurgy can achieve near-net shaping, it typically requires high sintering temperatures and long dwelling times [21,22], often leading to excessive grain growth and compromised mechanical properties [23,24]. Furthermore, Ta has a strong affinity for interstitial elements like oxygen (O) and nitrogen (N), and its mechanical properties, especially ductility, are extremely sensitive to even minor contamination during high-temperature processing [25,26]. Therefore, developing a novel preparation technique that can achieve full densification while effectively inhibiting grain coarsening and controlling impurity pickup is of paramount importance for advancing the application of pure Ta.

Spark plasma sintering (SPS), also known as pulsed electric current sintering (PECS), has emerged as a revolutionary powder consolidation technique [27,28]. Its unique features, including rapid heating rates, application of external pressure, and pulsed DC current, enable densification at comparatively lower temperatures and within shorter timeframes than conventional methods [29,30]. This process effectively suppresses grain growth, making it particularly suitable for preparing fine-grained or even nanostructured refractory metals [31,32]. Despite these significant advantages, the SPS technique also presents certain limitations [33,34]. The high equipment cost and the constraints imposed by the graphite die system typically limit the size and geometry of the as-sintered samples, hindering the fabrication of large or complex-shaped components. Furthermore, potential contamination from the graphite tools, especially carbon diffusion into the tantalum matrix at elevated temperatures, warrants careful consideration for high-purity applications. Additionally, accurate temperature measurement and control can be challenging, and the specific contributions of the electric field effects to densification and grain growth mechanisms remain active topics of scientific debate [35,36]. Therefore, a thorough understanding and optimization of the sintering parameters are crucial to harness the benefits of SPS while mitigating its drawbacks.

Previous studies have successfully demonstrated the capability of SPS to produce high-quality refractory alloy components. For instance, research on Ta-10W alloy has shown that optimizing parameters such as temperature, pressure, and holding time can yield refined microstructures and enhanced mechanical properties [37]. However, the research focus has predominantly remained on alloy systems. There remains a relative scarcity of systematic studies dedicated to the SPS process of pure Ta. The optimization of sintering parameters and their intricate interplay with the final microstructure and properties—such as density, grain size, oxygen content, and resultant strength and ductility—for pure Ta are not yet fully understood. Therefore, building upon the existing knowledge of SPS for refractory alloys, this work aims to systematically investigate the fabrication of high-performance pure Ta using the SPS technique. The specific objectives include (1) exploring the effects of key sintering parameters, including temperature, pressure, and holding time, on the densification behavior; (2) characterizing the evolution of microstructure (grain size, texture, porosity); (3) evaluating the resultant mechanical properties (microhardness, bending strength); and (4) establishing the process–property relationships to identify the optimal SPS window for achieving fully dense, fine-grained pure Ta with superior mechanical performance for advanced applications.

## 2. Experimental Procedure

The pure Ta powder with a purity of 99.9 wt.% was supplied by Ningxia Orient Tantalum Industry Co., Ltd., Shizuishan, China. The consumables, such as carbon paper and graphite dies, were supplied by Yuli Graphite Products Co., Ltd. (Huixian, China). The preparation sintering was carried out using a SPS system (LABOX-350, Sinterland, Niigata, Japan). The experimental design systematically investigated three key parameters across seven distinct conditions: sintering temperature (1350, 1400 and 1450 °C), applied pressure (20, 30 and 40 MPa), and holding time (2, 5 and 10 min), as comprehensively detailed in Table 1. The sintering procedure involved encapsulating the powder in carbon paper within a graphite die, applying a pre-pressure assembly to ensure proper powder packing and contact, followed by heating to the target temperature at 100 °C/min under a specified uniaxial pressure in a vacuum environment (≤10 Pa), and maintaining the conditions for the designated duration. All samples were subsequently furnace-cooled to room temperature, resulting in cylindrical compacts with a diameter of 20 mm and a height of 4 mm. The sintered cylindrical compact was cut by wire electrical discharge machining (WEDM). Longitudinal strips were sectioned for three-point bending tests, while the residual portions were mounted, ground, and polished to produce specimens for microstructural characterization and Vickers hardness measurement.

The density of the samples was measured using the Archimedes’ method in deionized water, and the relative density was calculated based on the theoretical density. Phase composition was analyzed by X-ray diffraction (XRD, Cu-Kα radiation, Smartlab, Rigaku, Tokyo, Japan) over a scanning range of 30°~90°. The surface and fracture morphology were observed using field-emission scanning electron microscope (FE-SEM, YF-1801, Yidong Optical Technology, Suzhou, China) and thermionic-emission scanning electron microscope (TE-SEM, Essence, Tescan, Brno, Czech Republic) in secondary electron (SE) detector mode. Phase composition analysis was performed using an energy dispersive spectrometer (EDS, X-Max 50, Oxford Instruments, Abingdon, UK) attached to the FE-SEM. Fracture surface morphology was examined using FE-SEM (YF-1801, Yidong Optical Technology, Suzhou, China) operating in InLens detector mode. The InLens mode, which provides high-resolution surface topography by collecting low-energy electrons directly from the specimen surface, was utilized to enhance contrast for analyzing fine microstructural details. Glow discharge mass spectrometry (GDMS, ELEMENT GD, Thermo Fisher Scientific, WA, USA) was used to determine the content of impurity elements. The average grain size of sintered specimens was counted from the fracture morphology using “nano measure” software (Version 2.1.0, MicroNano Measure Tech Co., Ltd., Guangzhou, China), and at least 100 grains were measured for each specimen.

Vickers hardness was measured under a load of 9.807 N with a dwell time of 15 s in accordance with ASTM E384 standard [38]. The bending strength was evaluated via a three-point bending test using non-standard specimen dimensions of 4 mm (width) × 2 mm (height) × 16 mm (length), with a span of 12 mm and a loading rate of 0.5 mm/min. The use of non-standard geometry was necessitated by the size constraints of the SPS-derived compacts. Although this limits direct comparability with absolute strength values reported in the literature for standard-sized specimens, it provides a rigorous basis for the relative comparison of the effects of sintering parameters within this study, as all tests were performed under identical geometrical conditions. Density, hardness, and bending strength values were averaged from at least five measurements.

## 3. Results and Discussion

### 3.1. Original Powder Analysis

**Figure 1:**
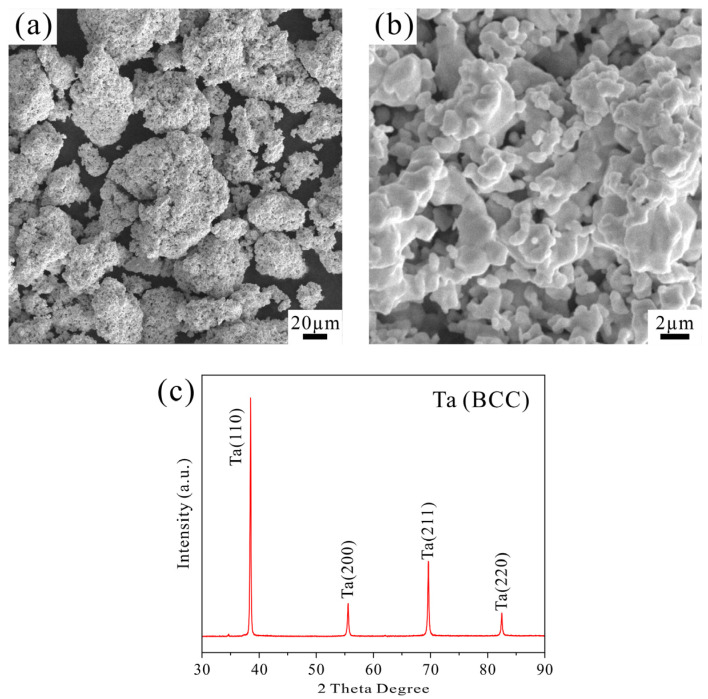
(**a**) SEM image of the Ta powder; (**b**) magnified view of the Ta powder; (**c**) XRD pattern of the Ta powder.

The microscopic morphology of the Ta powder is shown in Figure 1a, where significant agglomeration can be observed. This agglomerated structure likely originates from the preparation process, wherein excessively high reduction temperatures or prolonged dwelling time leads to the formation of partial sintering necks between powder particles, resulting in hard agglomeration. A magnified view of the Ta powder (Figure 1b) reveals an ellipsoidal morphology with particle sizes on the scale of several micrometers. The XRD pattern of the Ta powder is presented in Figure 1c, showing four diffraction peaks corresponding to the (110), (220), (211), and (200) crystal planes of Ta with a BCC structure. The XRD result indicates that the Ta powder exhibits high purity, with no detectable secondary phases or impurities such as oxides.

### 3.2. Sample Density Analysis

**Figure 2:**
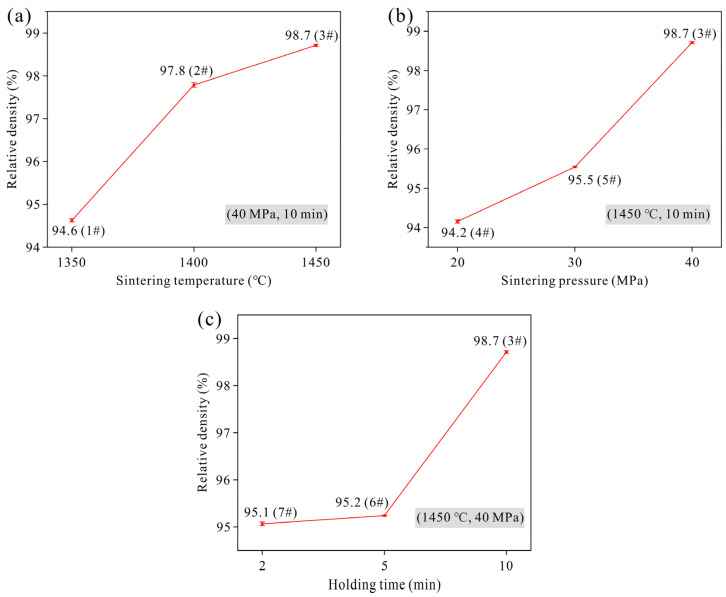
Variation in relative density of Ta samples under different sintering parameters: (**a**) sintering temperature (Samples 1#, 2# and 3#); (**b**) sintering pressure (Samples 4#, 5# and 3#); (**c**) holding time (Samples 7#, 6# and 3#).

The variation in relative density of Ta samples under different sintering parameters is shown in Figure 2. The results indicate that the relative density of the samples exhibits an increasing trend with the rise in sintering temperature, sintering pressure, and holding time. Under the conditions of a sintering pressure of 40 MPa and a holding time of 10 min, the relative density of the sample is 94.6% at a sintering temperature of 1350 °C. When the temperature increases to 1400 °C, the relative density significantly improves to 97.8%. A further temperature increase to 1450 °C results in a relative density of 98.7%. Within the temperature range of 1350~1450 °C, the relative density initially increases rapidly with rising temperature and then slows down. Similarly, increasing the sintering pressure and prolonging the holding time also contributes to the densification of the Ta powder. However, within the parameter range set in this study, the relative density shows an initial slow increase followed by an accelerated improvement with the enhancement of these two parameters. In comparison, the sintering temperature has the most pronounced effect on the densification behavior.

### 3.3. Phase Characterization and Microstructure Analysis

**Figure 3:**
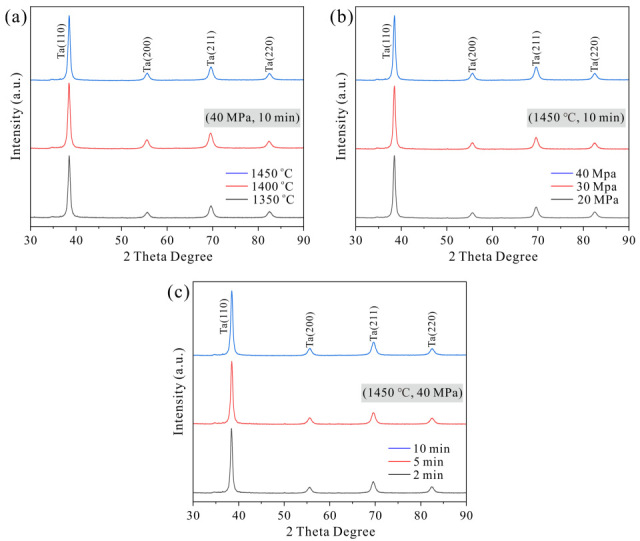
XRD patterns of three sets of Ta samples from comparative experiments: (**a**) different sintering temperature; (**b**) different sintering pressure; (**c**) different holding time.

XRD patterns of three sets of Ta samples from comparative experiments are displayed in Figure 3. The results indicate that the sintered Ta samples retains a BCC structure, with no detectable secondary phases or impurities such as oxides. Compared to the XRD pattern of the original Ta powder, the diffraction peaks of the sintered samples exhibit significant broadening, which is primarily attributed to grain refinement and microstrain induced during the sintering process. The grain refinement results from the relatively short duration of the SPS process, which prevents sufficient grain growth, yielding a smaller average size in the sintered bulk compared to the original particles. Microstrain arises from two primary factors: thermal residual stress caused by heterogeneous cooling and thermal expansion mismatch during post-sintering cooling, and processing stress resulting from plastic deformation during powder compaction and sintering, which introduces dislocations, point defects, and other crystalline defects that distort the lattice. The combined effect of these mechanisms leads to the observed diffraction peak broadening.

**Figure 4:**
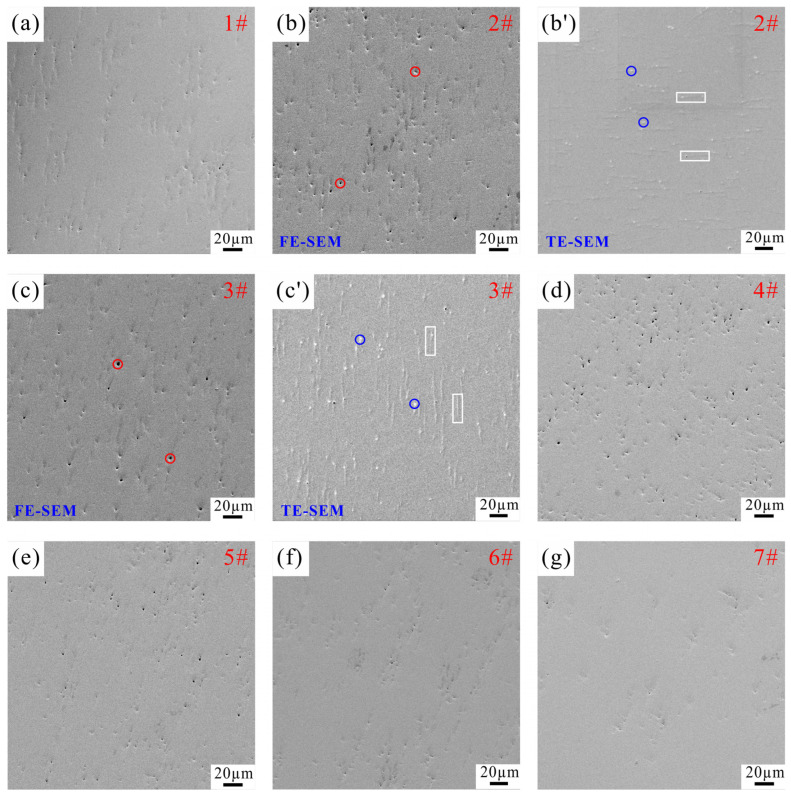
SEM images of samples prepared under different sintering processes: (**a**) Sample 1; (**b**,**b′**) Sample 2; (**c**,**c′**) Sample 3; (**d**) Sample 4; (**e**) Sample 5; (**f**) Sample 6; (**g**) Sample 7.

Figure 4 shows the SEM images of samples prepared under different sintering processes, which are acquired using FE-SEM operating in SE mode. The micrographs reveal that all samples consist of a single-phase structure with no detectable impurity phases or oxides, which is consistent with the XRD analysis results. Notably, apparent pore-like structures (indicated by red circles) are observed in Figure 4b,c (corresponding to Samples 2 and 3), whereas such features are absent in Figure 4f,g (Samples 6 and 7). These observations appear contradictory to the relative density data presented in Figure 2, which indicate that Samples 2 and 3 exhibit significantly higher density than Samples 6 and 7. To investigate this discrepancy, further characterization is conducted on Samples 2 and 3 using TE-SEM under SE mode, as shown in Figure 4b′,c′. Comparative analysis confirms that the suspected pores are actually artifacts introduced during polishing—referred to as “pseudo-pores.” Due to the higher hardness of Samples 2 and 3, abrasive particles (such as diamond suspension) tend to roll and drag across the surface rather than embed into the matrix, resulting in micron-scale scratches (highlighted with white rectangles). Under SE imaging, these scratches appear as dark troughs resembling pores, while material accumulation at scratch intersections forms bright protrusions (marked by blue circles), enhancing the contrast of the depressed regions and leading to misinterpretation as pores.

**Figure 5:**
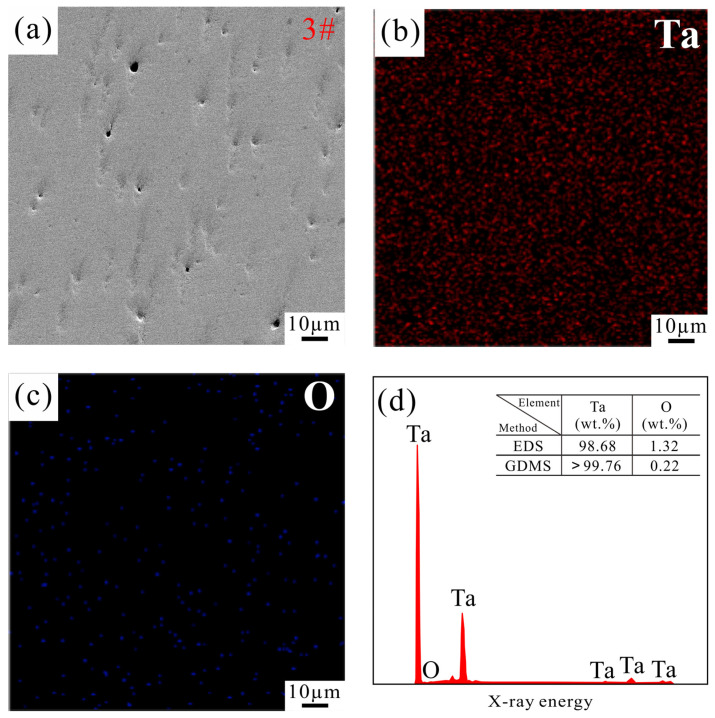
(**a**) SEM micrographs of Sample 3; (**b**,**c**) surface EDS analysis of elements Ta and O; (**d**) EDS and GDMS results of sample 3.

Figure 5 exhibits the SEM micrographs and the corresponding surface elemental analysis results of Sample 3. Oxygen is regarded as a key impurity in metal sputtering targets owing to its propensity for forming particulate inclusions in thin films, potentially inducing circuit shorting and thus degrading device performance [39,40]. As shown in Figure 5b,c, the elements are uniformly distributed on the sample surface, with Ta being the primary constituent and a relatively weak signal intensity of oxygen. EDS analysis confirms that the characteristic peaks correspond predominantly to Ta, while the oxygen-related peaks are barely detectable. Quantitative results indicate an O content of 1.32 wt.%. Furthermore, GDMS analysis demonstrates a significantly lower oxygen content of only 0.22 wt.%. These consistent findings reveal that the relatively low sintering temperature and short holding time employed in the SPS process effectively suppress the oxidation of the Ta powder.

**Figure 6:**
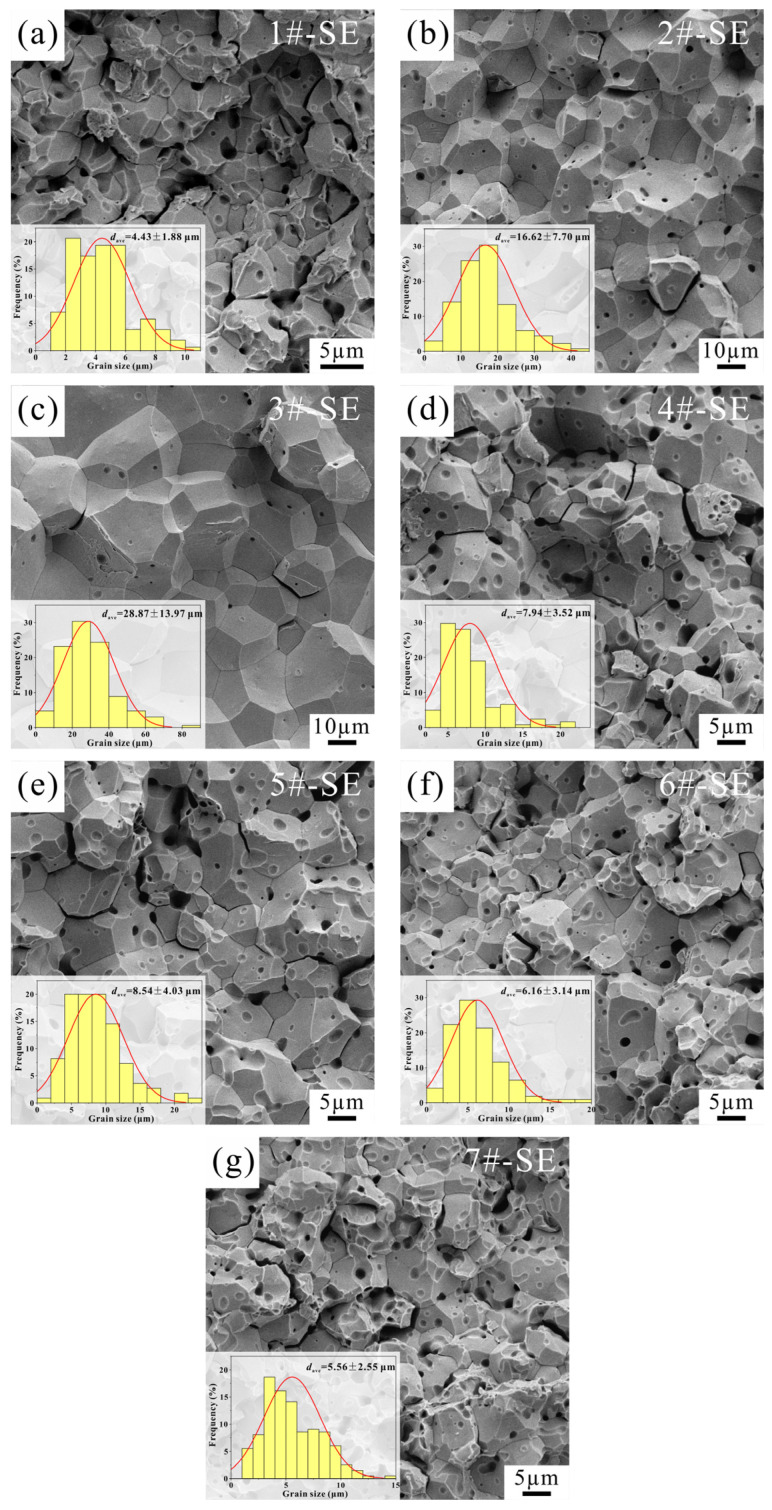
Fracture morphology and corresponding grain size distribution of samples prepared under different sintering processes: (**a**) Sample 1; (**b**) Sample 2; (**c**) Sample 3; (**d**) Sample 4; (**e**) Sample 5; (**f**) Sample 6; (**g**) Sample 7.

**Figure 7:**
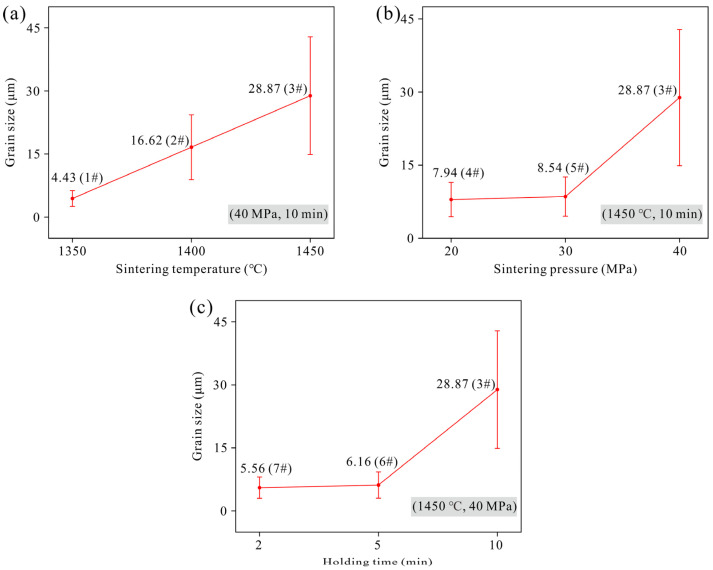
Influence of various sintering parameters on the grain sizes of the specimens: (**a**) sintering temperature (Samples 1#, 2# and 3#); (**b**) sintering pressure (Samples 4#, 5# and 3#); (**c**) holding time (Samples 7#, 6# and 3#).

The fracture morphology and corresponding grain size distribution of Ta specimens observed under SE mode after different sintering processes is presented in Figure 6. Figure 7 summarizes the influence of various sintering parameters on the grain sizes of the specimens. The results indicate that as the sintering temperature, sintering pressure, or holding time increases, the grain size generally exhibits an increasing trend. Under the conditions of a sintering pressure of 40 MPa and a holding time of 10 min, when the sintering temperature is 1350 °C, the average grain size of the specimen is 4.43 μm, with numerous micropores visible in the fracture surface (Figure 6a). As the sintering temperature rises to 1400 °C and 1450 °C, the grain size increases significantly, reaching average sizes of 16.62 μm and 28.87 μm (Figure 6b,c), respectively, indicating a positive correlation between sintering temperature and grain size. In addition, almost no pores are observed in the specimens sintered at higher temperatures, suggesting higher densification, which is consistent with the relative density values of Sample 2 (97.8%) and Sample 3 (98.7%) being significantly higher than that of Sample 1 (94.6%). From Figure 7b,c, it can be seen that the grain size increases slowly at first and then more rapidly with increasing sintering pressure and holding time. This trend reflects differences in the densification mechanisms of the powder at different sintering stages. According to the Li et al. [41], the powder sintering process can be divided into an initial stage dominated by densification and a later stage dominated by grain growth. In this study, the sintering behavior of the samples with relatively low relative density (Samples 1, 4, 5, 6, and 7, with densities between 94.2% and 95.5%) is predominantly controlled by the densification process. When the relative density exceeds a threshold value (inferred to be approximately 96% in this work), as in Samples 2 and 3, the sintering process is governed by a combined mechanism of densification and grain growth.

### 3.4. Mechanical Properties and Fracture Analysis

**Figure 8:**
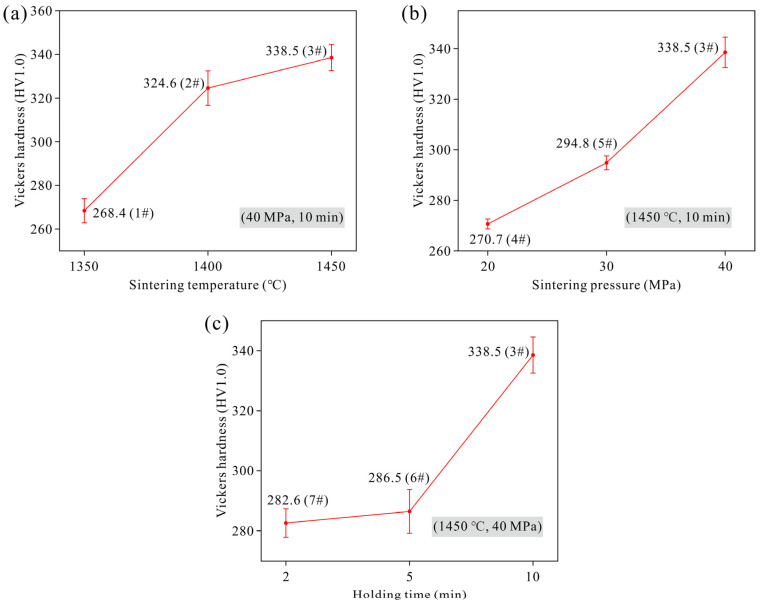
Variation in hardness of Ta samples under different sintering parameters: (**a**) sintering temperature (Samples 1#, 2# and 3#); (**b**) sintering pressure (Samples 4#, 5# and 3#); (**c**) holding time (Samples 7#, 6# and 3#).

Figure 8 shows the hardness test results of sintered specimens from three sets of controlled experiments. It can be observed that the hardness of the specimens exhibits a clear consistent trend with the relative density: higher relative density corresponds to greater hardness. Specifically, Sample 3 possesses the highest relative density (98.7%) and also achieves the maximum hardness value of 338.5 HV1. In contrast, Samples 1 (94.6%), 4 (94.2%), and 7 (95.1%) show lower relative densities, and their hardness values are correspondingly lower, ranging from 268.4 HV1 to 282.6 HV1.

**Figure 9:**
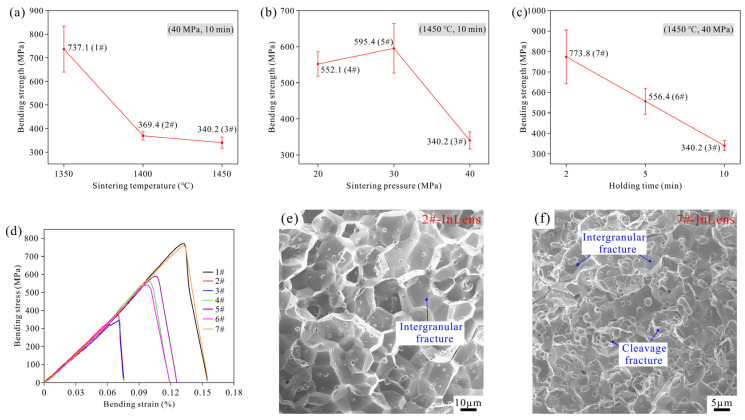
(**a**–**c**) Bending strength test results of sintered specimens from three control groups: (**a**) sintering temperature (Samples 1#, 2# and 3#); (**b**) sintering pressure (Samples 4#, 5# and 3#); (**c**) holding time (Samples 7#, 6# and 3#); (**d**) bending stress–strain curves; (**e**,**f**) fracture morphology of Samples 2# and 7#.

Figure 9a–c presents the bending strength test results of sintered specimens from three control groups. It can be observed that the bending strength decreases significantly with increasing sintering temperature and holding time, while it first increases slightly and then decreases rapidly with increasing sintering pressure. Specifically, Samples 1 (1350 °C/40 MPa/10 min) and 7 (1450 °C/40 MPa/2 min) exhibit higher bending strengths of 737.1 MPa and 773.8 MPa, respectively. In contrast, Sample 2 (1400 °C/40 MPa/10 min) and 3 (1450 °C/40 MPa/10 min) show lower bending strengths of 369.4 MPa and 340.2 MPa, respectively. Figure 9d shows the bending stress–strain curves of the specimens from the three control groups. The results indicate that specimens with higher bending strength generally demonstrate greater plastic deformation capability. Based on the overall performance in strength and plasticity, the specimens can be categorized into three distinct levels: Samples 1 and 7 exhibit the best performance, followed by Samples 4–6, while Samples 2 and 3 show the poorest performance. To investigate the underlying mechanisms, the fracture surfaces of Samples 2 and 7 are presented in Figure 9e,f. It is evident that Sample 2 exhibits coarse grains with intergranular fracture being the dominant failure mechanism. In comparison, Sample 7 displays finer grains and a mixed fracture mode comprising both intergranular and cleavage fractures. These findings demonstrate that the fracture mechanism plays a critical role in determining the bending strength and plastic deformation behavior of the specimens.

## 4. Conclusions

In summary, this study has successfully prepared high-density pure Ta with excellent mechanical properties using SPS by systematically varying temperature, pressure, and holding time. The main conclusions are as follows:

(1) Increasing the sintering temperature, pressure, and holding time all contribute to enhancing the densification process of pure Ta, among which the sintering temperature exhibits the predominant effect. Under the process conditions of 1450 °C, 40 MPa, and 10 min, the sample achieves the highest relative density of 98.7%. This study successfully controls the oxygen content at a low level, which facilitates the production and application of high-purity Ta sputtering targets.

(2) The hardness of the samples shows a positive correlation with their relative density. The sample sintered at 1450 °C/40 MPa/10 min exhibits the highest hardness, reaching 338.5 HV1. In contrast, the bending strength of the samples is negatively correlated with grain size. The sample sintered at 1450 °C/40 MPa/2 min demonstrates the maximum bending strength of 773.8 MPa. As the sintering temperature, pressure, and holding time increase, grain coarsening occurs, causing the fracture mechanism to transition from a mixed mode of cleavage and intergranular fracture to entirely intergranular fracture. This transition results in a decrease in both bending strength and plastic deformation capacity.

Beyond the specific findings on parameter optimization, this study demonstrates the significant potential of the SPS technique in fabricating high-performance pure tantalum. The achieved high density and fine-grained microstructure under relatively lower temperatures highlight a promising route for energy-efficient production of refractory metals. These findings have direct practical implications for manufacturing high-purity tantalum sputtering targets with enhanced performance for the semiconductor industry. Future research will focus on further refining the SPS process window, exploring the effects of Ta powder characteristics, and extending this methodology to fabricate advanced Ta-based alloys for extreme environment applications.

## Figures and Tables

**Table 1:** The process parameters of SPS.

Sample Number	Sintering Temperature/ °C	Sintering Pressure/ MPa	Holding Time/ min
1	1350	40	10
2	1400	40	10
3	1450	40	10
4	1450	20	10
5	1450	30	10
6	1450	40	5
7	1450	40	2

## Data Availability

The original contributions presented in this study are included in the article. Further inquiries can be directed to the corresponding authors.

## Abbreviations

**BCC**	Body-Centered Cubic
**EBM**	Electron Beam Melting
**EDS**	Energy Dispersive X-ray Spectroscopy
**FE-SEM**	Field-Emission Scanning Electron Microscope
**GDMS**	Glow Discharge Mass Spectrometry
**PVD**	Physical Vapor Deposition
**SE**	Secondary Electron
**SEM**	Scanning Electron Microscope
**SPS**	Spark Plasma Sintering
**TE-SEM**	Therminoic-Emission Scanning Electron Microscope
**WEDM**	Wire Electrical Discharge Machining
**XRD**	X-ray Diffraction
